# Primary ALK-negative ALCL of the small intestine: a rare case report with review of literature

**DOI:** 10.1007/s13691-025-00787-6

**Published:** 2025-07-29

**Authors:** Tanisha Singla, Nilay Nishith, Rahul Raj, Aishwarya Sharma, Puneet Kaur Somal, Ravikiran Pawar, Sankalp Sancheti, Simran Kalra

**Affiliations:** 1 Department of Onco-Pathology, Homi Bhabha Cancer Hospital and Research Centre, Tata Memorial Centre, Homi Bhabha National Institute (HBNI), New Chandigarh, Punjab India; 2 Department of Nuclear Medicine, Homi Bhabha Cancer Hospital and Research Centre, Tata Memorial Centre, Homi Bhabha National Institute (HBNI), New Chandigarh, Punjab India

**Keywords:** ALK-1, ALCL, Diagnosis, Small intestine, Rare

## Abstract

ALK-negative primary anaplastic large cell lymphoma (ALCL) of the small intestine is exceptionally rare and presents significant diagnostic and therapeutic challenges. Characterized by large-sized neoplastic lymphoid cells with scant cytoplasm and pleomorphic nuclei, its clinical presentation is often nonspecific, mimicking infections or inflammatory disorders. We present a rare case of ALK-negative primary ALCL of the small intestine in a 35-year-old male who presented with fever, abdominal pain, and significant weight loss. This case highlights the need for heightened clinical suspicion, a comprehensive histopathological and immunophenotypic approach, and optimal management strategies for this aggressive entity.

## Introduction

Anaplastic large cell lymphomas (ALCLs) are mature T-cell lymphomas classified into ALK-positive, ALK-negative, and breast implant-associated ALCLs. While they share histomorphologic and immunophenotypic features, they are clinically and genetically distinct. ALK-negative ALCLs are defined by genetic alterations such as DUSP22-IRF4 and TP63 rearrangements, as well as JAK/STAT pathway mutations [1]. Additionally, they can involve both nodal and extranodal sites, with ALK-negative ALCLs affecting lymph nodes and extranodal sites in a 1:1 ratio. Common extranodal locations include the skin, soft tissue, liver, and spleen, whereas gastrointestinal (GI) involvement is rare [1]. Primary ALCL of the small intestine is an exceedingly uncommon entity, with ALK-negative cases being even rarer. Most cases present with nonspecific symptoms such as abdominal pain, weight loss, and bowel obstruction, often mimicking tuberculosis or enteric fever [2].

Herein, we present a case of ALK-negative primary ALCL of the small intestine in a 35-year-old male who presented with fever, abdominal pain, and significant weight loss. This case adds to the limited literature on primary ALK-negative ALCL of the small intestine, particularly from regions like the Indian subcontinent. Beyond geographic novelty, this case provides valuable insights into the diagnostic challenges of distinguishing ALK-negative ALCL from more common gastrointestinal lymphomas in resource-limited settings and illustrates the application of novel therapeutic approaches with CD30-targeted therapy in this rare entity.

## Case report

**Fig. 1:**
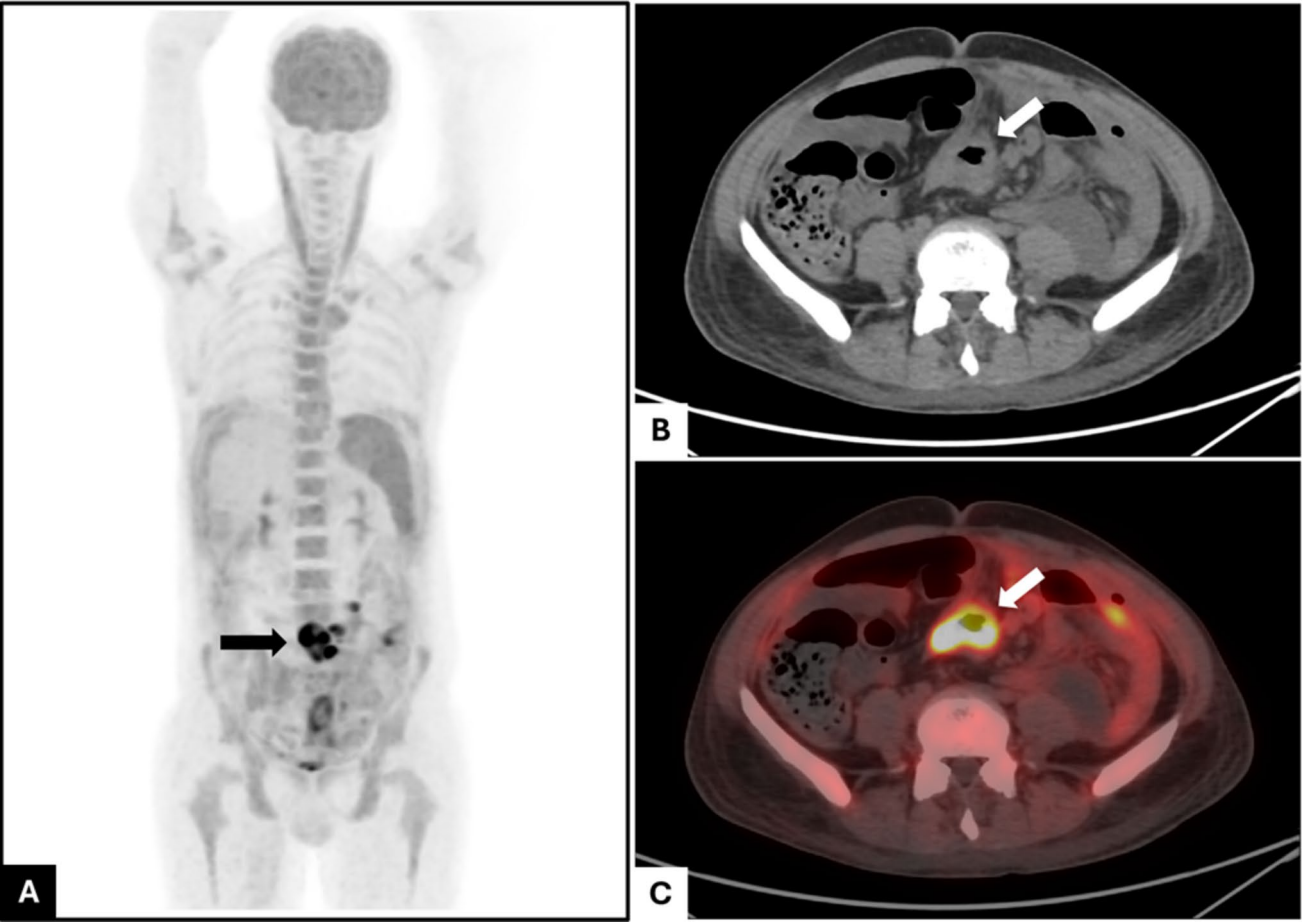
PET-CT whole body **A** Maximum intensity projection (MIP) image showing a focus of increased tracer uptake in small bowel (back arrow; coronal plane). **B**, **C** CT and PET-CT fusion images showing circumferential thickening in small bowel with increased 18F-FDG uptake (white arrow, transverse plane)

A 35-year-old male presented with a 15-day history of fever, abdominal pain, and significant weight loss. Given the high prevalence of infectious diseases in India, initial differentials included tuberculous enteritis and enteric fever. His preliminary hematological, biochemical, and serological investigations were largely unremarkable, except for severe anemia and elevated lactate dehydrogenase (LDH) levels. A positron emission tomography-computed tomography (PET-CT) scan revealed multiple fluorodeoxyglucose (FDG)-avid, thickened small bowel segments (SUVmax: 21.79) without significant lymphadenopathy elsewhere. Additionally, multiple low-grade FDG-avid peritoneal nodules (SUVmax: 7.84) were identified (Fig. 1). During the initial investigation, the patient's condition deteriorated as he developed pneumoperitoneum. This necessitated an exploratory laparotomy, followed by jejunal resection with J-J anastomosis, which revealed perforations.

**Fig. 2:**
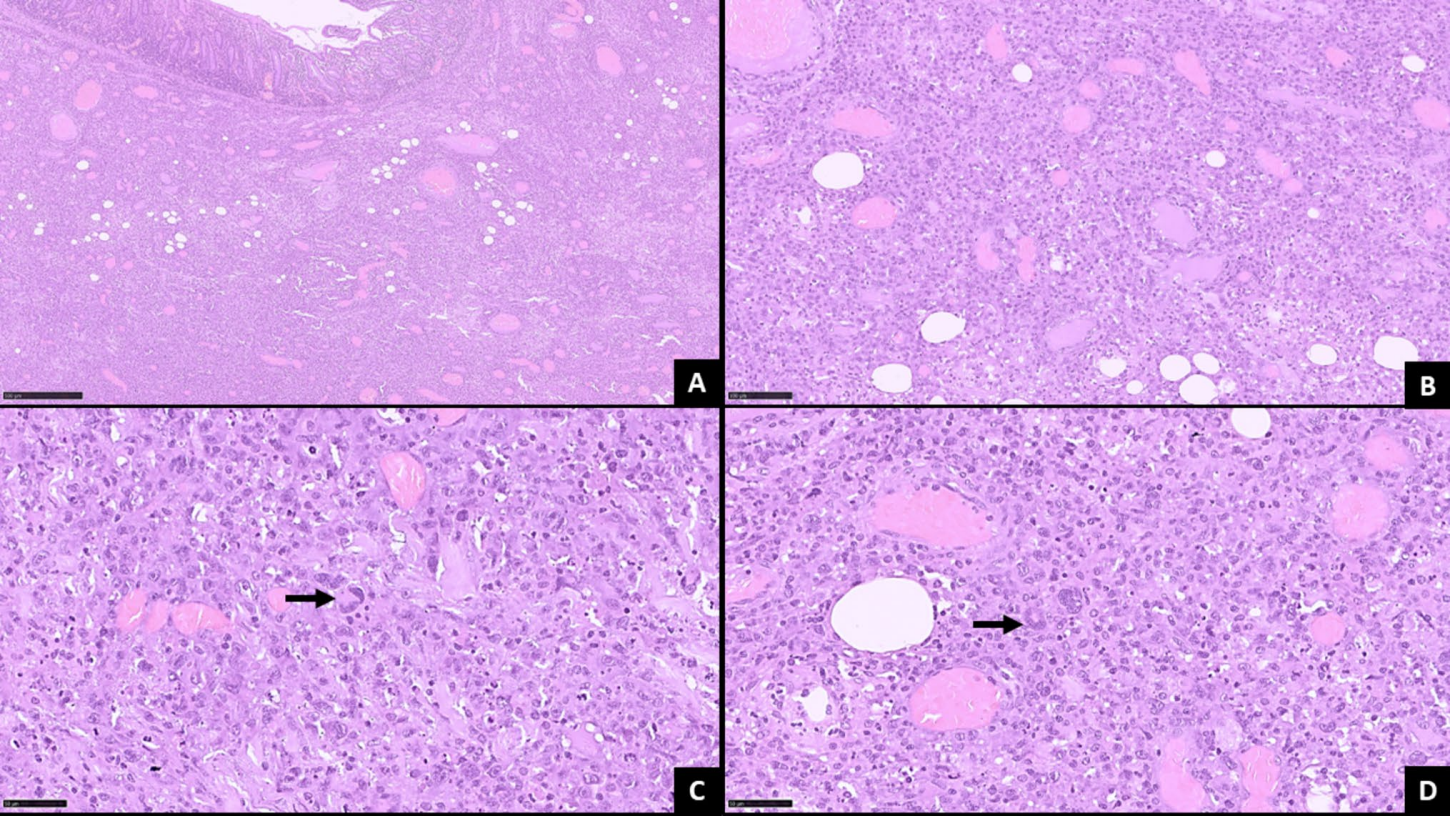
**A**–**D** Histopathological findings. **A**. Scanner view showing diffuse infiltration of the intestinal wall by atypical lymphoid cells (H&E, ×50). **B**. Sheets of large-sized atypical lymphoid cells exhibiting marked nuclear pleomorphism, vesicular chromatin, prominent nucleoli, irregular nuclear contour, and scant cytoplasm (H&E, ×200). **C**. High magnification showing characteristic Hallmark cells indicated by a black arrow (H&E, ×400). **D**. High magnification showing characteristic doughnut/wreath-like cells indicated by a black arrow (H&E, ×400)

**Fig. 3:**
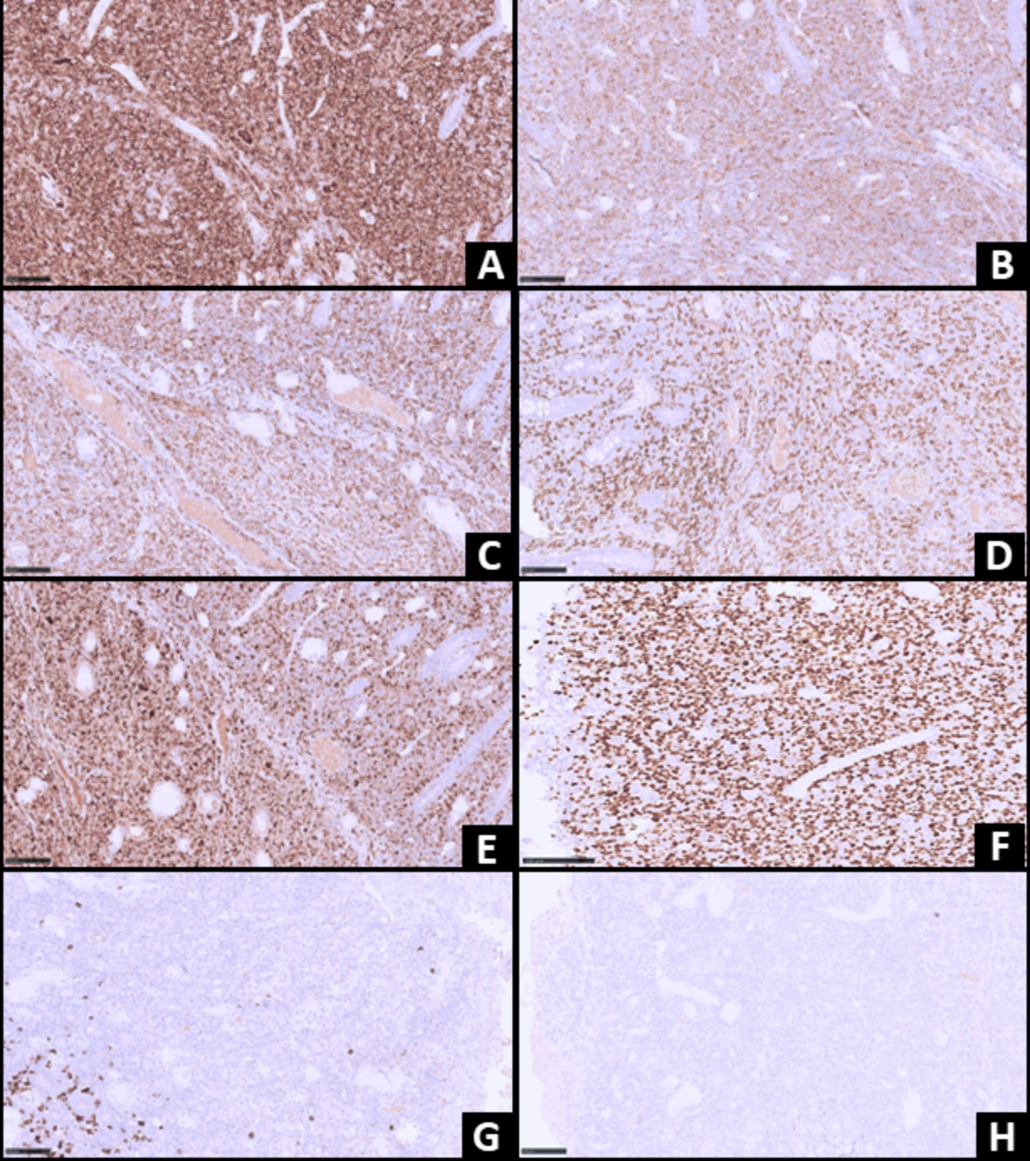
**A**–**H**: The atypical lymphoid cells exhibiting positivity for CD30 [200x] (**A**), EMA [200x] (**B**), CD7 [200x] (**C**), CD4 [200x] (**D**), Granzyme B [200x] (**E**), MUM1 [200x] (**F**); while they were negative for CD20 [200x] (**G**) and ALK1 [200x] (**H**)

Gross examination of the resected specimen (wedge resection of jejunum) revealed a markedly thickened intestinal wall with multiple mucosal ulcers and diffuse exudate on the serosal surface. Microscopic evaluation showed diffuse infiltration of the intestinal wall by large atypical lymphoid cells with pleomorphic vesicular nuclei, prominent nucleoli, irregular nuclear contour, and scant cytoplasm. Characteristic hallmark cells and doughnut cells were observed, along with numerous mitotic figures and frequent apoptotic bodies (Fig. 2). Immunohistochemical (IHC) staining was performed using a comprehensive panel of antibodies to characterize the cells. The panel included CD30 (Ber-H2, Cell Marque), EMA (BGA M0410, Bioheaven 360), MUM1 (MUM1p, Dako), CD7 (LP15, Biocare), CD4 (SP35, Ventana), CD8 (SP57, Ventana), CD20 (L26, Zeta), CD3 (Rabbit Polyclonal, Cell Marque), CD5 (IHC738, Genome me), CD10 (SP67, Ventana), BCL2 (124, Dako), BCL6 (G/191E, Cell Signaling), c-MYC (IHC448, Genome me), ALK-1 (ALK1, Dako), and MIB-1 (8D5, Cell Signaling). Ready-to-use (RTU) dilutions were utilized for EMA, MUM1, CD7, CD4, CD8, CD5, CD10, BCL2, c-MYC, and ALK-1. Specific dilutions were used for CD20 (1:250), CD3 (1:250), BCL6 (1:1200), and MIB-1 (1:2000). The immunohistochemical panel demonstrated strong, diffuse positivity for CD30, EMA, MUM-1, CD7, CD4 (CD4 > CD8), and Granzyme B in the atypical lymphoid cells. The neoplastic cells were negative for CD20, CD3, CD5, CD10, BCL-2, BCL-6, and ALK-1 (Fig. 3). The MIB-1 proliferation index was approximately 90%, indicating a highly proliferative neoplasm. Based on radiological, histomorphological, and immunohistochemical findings, a diagnosis of primary ALK-negative anaplastic large cell lymphoma of the jejunum was established. Molecular analysis for DUSP22 and TP63 rearrangements or JAK/STAT pathway mutations was not performed due to limited resources.

**Fig. 4:**
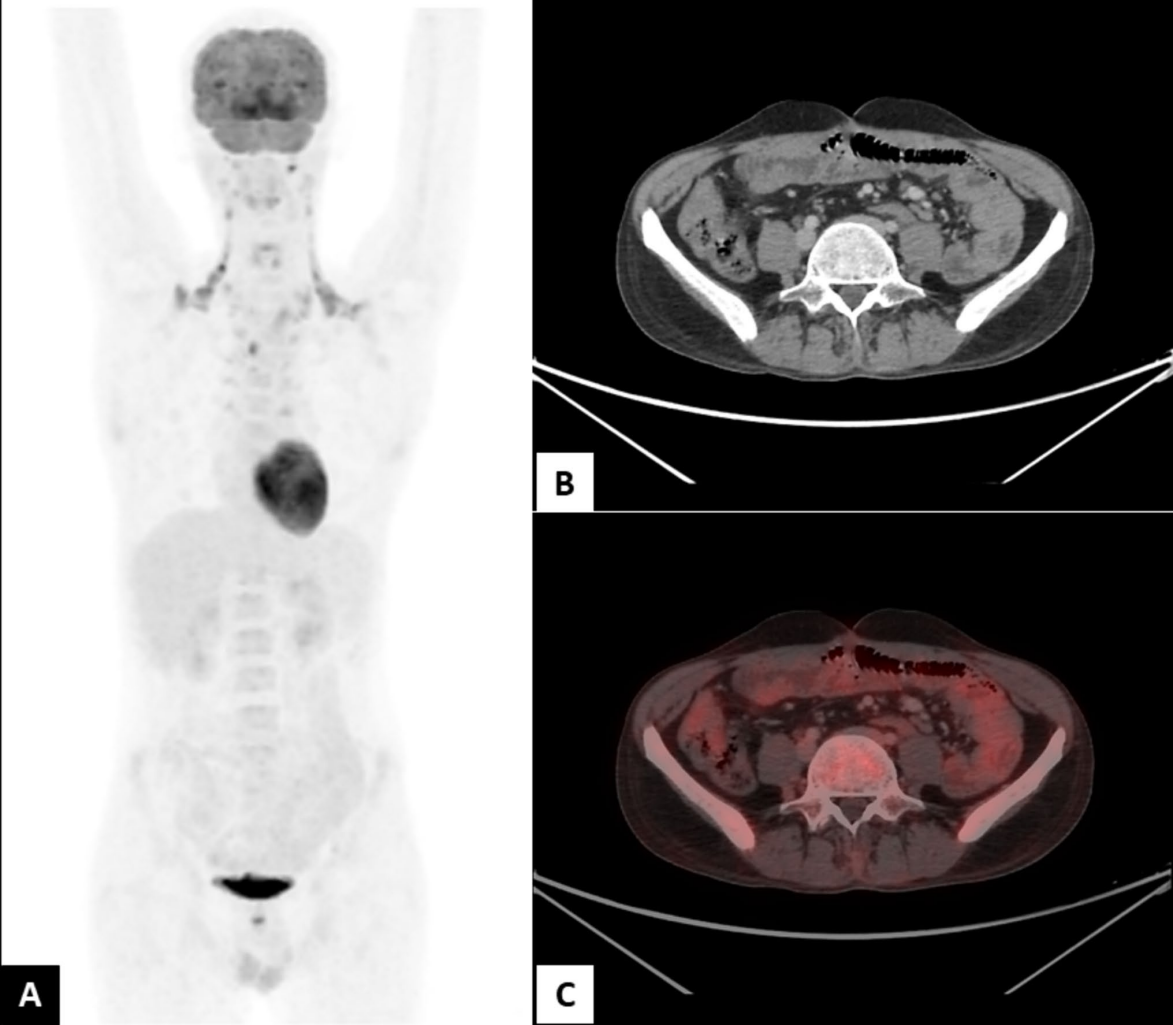
**A**–**D**: Post-chemotherapy images show complete resolution of previous known disease. MIP image shows brown fat expression in the bilateral nape of neck, which is physiological

Clinically, the patient was classified as stage IVBE (extranodal) with an International Prognostic Index (IPI) score of 4. Treatment was initiated with the BV-CHP chemotherapy regimen (Brentuximab Vedotin, Cyclophosphamide, Doxorubicin, and Prednisolone) for six cycles, along with granulocyte colony-stimulating factor (G-CSF) support. Six months have passed since the initial diagnosis. The patient has completed five cycles of chemotherapy. A PET scan performed following the fourth cycle demonstrated no evidence of metabolically active disease. There is a complete metabolic and morphological resolution of all previously identified lesions (Fig. 4).

## Discussion

Primary ALCL of the small intestine is exceptionally rare, with ALK-negative ALCL being even more uncommon—fewer than 20 cases have been documented in the literature [2–5]. The clinicopathological features and follow-up details of all reported cases to date, along with the index case, have been summarized in Table 1. This table indicates 19 cases of ALCL with involvement of small intestine. The majority of patients were male (16/19) with a median age in the mid-50s. Common primary sites included the jejunum, small intestine, duodenum, and ileum. Frequent symptoms were abdominal pain, distension, and mass, with bowel perforation noted in 10 of 16 reported cases. All cases were CD30-positive and ALK-1 negative, often showing EMA, TIA1, Granzyme B, and MUM1 positivity. Treatment typically involved surgical resection, frequently followed by CHOP chemotherapy. Outcomes varied, with many patients dying from disease or complications like sepsis, while survival ranged from weeks to over 60 months.

**Table 1:** Clinicopathological characteristics and outcomes of reported cases of primary ALK-negative ALCL of the small intestine

Case	Age (in years)/Sex	Location	Symptoms	IHC Markers	Stage	Perforation Status	Treatment	Outcome	Survival	References
1	72/M	Jejunum	Diffuse abdominal tenderness	CD30+, EMA+; CD3+, CD20-, CD43+; TIA1-; Perforin+; ALK 1-, EBVLMP-, CD15-, EBER-ISH-	-	Negative	Segmental resection	Died of sepsis	4 weeks	Carey et al. [5]
2	42/M	Duodenum and Jejunum	Abdominal cramps	CD30+, EMA-; CD3+, CD20-, CD43-; TIA1+; Perforin-; ALK 1-, EBVLMP+, CD15-, EBER-ISH+	-	Not done	Segmental resection; chemotherapy	Died of disease	8 months	Carey et al. [5]
3	77/M	Duodenum and Jejunum	Painless jaundice, pruritus	CD30+, EMA+; CD3+, CD20-, CD43+; TIA1+; ALK 1-, EBVLMP-, CD15-, EBER-ISH-	-	Negative	Whipple resection	Died of sepsis	6 weeks	Carey et al. [5]
4	65/M	Jejunum	Fever	CD30+, CD2+, Granzyme B+, EMA+; ALK 1-	IV E	Present	Segmental resection	Died	0.7 months	Lee et al. [4]
5	88/M	Terminal ileum	Not mentioned	CD30+, TIA-1+, Granzyme B+, MUM 1+; ALK 1-	II E	Negative	Segmental resection + CHOP	Alive with disease	4+ months	Lee et al. [4]
6	57/M	Terminal ileum	Not mentioned	CD30+, EMA+, Granzyme B+, MUM 1+; ALK 1-	I E	Not mentioned	Right hemicolectomy	Died	0.7 months	Lee et al. [4]
7	56/M	Duodenum	Not mentioned	CD3+, CD4+, CD30+, TIA1+, ALK 1-	-	Not mentioned	Surgery + Adjuvant chemo	Not mentioned	Not mentioned	Kim et al. [3]
8	50/M	Duodenum	Not mentioned	CD3+, CD4+, CD30+, TIA1+, ALK 1-	-	Not mentioned	Surgery + Adjuvant chemo	Not mentioned	Not mentioned	Kim et al. [3]
9	34/F	Small bowel	Not mentioned	CD3+, CD4+, CD30+, TIA1+, ALK 1-	-	Present	Surgery + Adjuvant chemo	Not mentioned	Not mentioned	Kim et al. [3]
10	22/M	Ileum	Abdominal pain, abdominal distension, abdominal mass	CD30+, EMA+, CD3-, ALK 1-,	IIE	Present	Resection + CHOP	Died	<12 months	Ud Din et al. [2]
11	50/F	Small intestine	Abdominal pain, abdominal distension	CD30+, EMA+, CD3-, ALK 1-	IIE	Present	Resection + CHOP	Died	Few months	Ud Din et al. [2]
12	49/M	Small intestine	Abdominal pain, abdominal mass	CD30+, EMA+, CD3+, ALK 1-	IE	Negative	Resection + CHOP	Alive	At 60 months	Ud Din et al. [2]
13	50/F	Duodenum	Abdominal pain, abdominal distension, abdominal mass	CD30+, EMA+, CD3-, ALK 1-	IE	Not mentioned	CHOP	Died	<12 months	Ud Din et al. [2]
14	52/M	Small intestine	Abdominal pain, abdominal distension	CD30+, EMA+, CD3+, ALK 1-	IIE	Negative	Resection + CHOP	Died	<3 months	Ud Din et al. [2]
15	35/M	Small intestine	Abdominal pain, abdominal mass	CD30+, EMA+, CD3+, ALK 1-	IV	Negative	Resection + CHOP	Died	6 months	Ud Din et al. [2]
16	65/M	Jejunum	Abdominal pain, abdominal distension, abdominal mass	CD30+, EMA+, CD3-, ALK 1-	IIE	Present	Resection	Died	few months	Ud Din et al. [2]
17	45/F	Ileum	Abdominal pain, abdominal mass	CD30+, EMA+, CD3+, ALK 1-	IE	Present	Resection + CHOP	Died	<12 months	Ud Din et al. [2]
18	59/M	Jejunum	Abdominal pain, abdominal distension, abdominal mass	CD30+, EMA+, CD3-, ALK 1-	IE	Negative	Resection	Died	<12 months	Ud Din et al. [2]
19	35/M	Jejunum	Fever, abdominal pain, significant weight loss	CD30+, EMA+, MUM-1+, CD7+, CD4+, Granzyme B +, CD20-, ALK 1-	IVBE	Present	Resection+ BV-CHP	Alive with resolution disease	Till date	Index case

Given its rarity, ALK-negative ALCL must be carefully distinguished from other more common gastrointestinal lymphomas, including classical Hodgkin lymphoma (CHL), diffuse large B-cell lymphoma (DLBCL), and various T-cell lymphomas such as enteropathy-associated T-cell lymphoma (EATL), monomorphic epitheliotropic intestinal T-cell lymphoma (MEITL), extranodal NK/T-cell lymphoma (ENKTCL), and intestinal T-cell lymphoma, NOS [2]. DLBCL is the most frequently occurring primary gastrointestinal lymphoma and should always be considered in the differential diagnosis. A subset of DLBCLs expresses CD30 and can exhibit anaplastic morphology, making it difficult to distinguish from ALK-negative ALCL [6]. However, the expression of CD20 in DLBCL serves as a critical differentiating marker. ALCL can sometimes mimic classical Hodgkin lymphoma morphologically, posing a diagnostic challenge. Rarely, weak PAX5 expression has been reported in both ALK-positive and ALK-negative ALCL, potentially leading to misclassification as CHL [7]. Hence, a comprehensive morphologic evaluation, along with an extensive immunophenotypic workup—including pan-T-cell markers—is essential for accurate diagnosis. Enteropathy-Associated T-Cell Lymphoma commonly involves the small intestine, but it was excluded in our case due to the absence of a history of celiac disease. Additionally, there was no histologic evidence of epitheliotropism, and the adjacent mucosa lacked features of celiac disease, such as villous atrophy, crypt hyperplasia, and intraepithelial lymphocytosis. Immunophenotypically, EATL is CD3 + and is double negative for CD4 and CD8, whereas our case was CD3-negative, with positivity of CD4 predominance over CD8 [2]. Monomorphic epitheliotropic intestinal T-cell lymphoma (MEITL) is a subtype of T-cell lymphoma that typically consists of small to medium-sized lymphoid cells with epitheliotropism and is immunophenotypically positive for CD8 but negative for CD4 and CD30 [8]. Given that our case was CD4-positive and lacked epitheliotropism, MEITL was ruled out. While ENKTCL can involve the small intestine in both nasal and non-nasal forms, it typically demonstrates angioinvasion and angiodestruction, which were not observed in our case. Additionally, ENKTCL often expresses CD30 and EMA, along with NK-cell markers (CD56) or T-cell markers (CD8), but lacks CD4 expression. The immunoprofile of our case (CD4 > CD8 and immune-negativity for CD56) did not match ENKTCL, further excluding this diagnosis [9]. Lastly, intestinal T-cell lymphoma, NOS are CD3-positive and may variably express CD30. However, ALCL is uniquely characterized by diffuse and strong CD30 positivity, with variable pan-T-cell antigen expression as was seen in our case [10].

ALK-negative ALCL has a distinct molecular signature compared to ALK-positive ALCL. While ALK-positive ALCL is defined by ALK rearrangements (commonly NPM1-ALK), ALK-negative ALCL is associated with DUSP22 (20–30%), TP63 (~ 5%), and JAK1/STAT3 mutations (~ 30%) [1]. Prognostically, DUSP22-rearranged cases have favourable outcomes, while TP63-expressing and triple-negative cases have worse prognoses [11]. Despite recognizing the prognostic importance of molecular subtypes in ALK-negative ALCL, fluorescence in situ hybridization (FISH) for DUSP22 and TP63 rearrangements and next-generation sequencing for JAK/STAT pathway mutations could not be performed due to resource constraints. This represents a limitation in our comprehensive assessment, as DUSP22-rearranged ALK-negative ALCLs demonstrate significantly better outcomes (5-year OS of 80%) compared to triple-negative cases (5-year OS of 33%) [12].

A review by Ferreri et al. highlighted the aggressive nature of ALK-negative ALCL, with most cases presenting at advanced stages, exhibiting B symptoms, high IPI scores, and elevated LDH levels [13]. Our patient, who had stage IV disease, an IPI score of 4, and elevated LDH, aligns with these findings.

The management of gastrointestinal ALK-negative anaplastic large cell lymphoma remains challenging, with CHOP (cyclophosphamide, doxorubicin, vincristine, prednisone) chemotherapy serving as the historical standard regimen [14]. However, outcomes are suboptimal, with complete response (CR) rates of 30–40% and median survival of 6–12 months, limited by hematological and cardiac toxicities that reduce compliance in elderly patients. Combined surgery and CHOP chemotherapy improve outcomes in localized disease, achieving CR rates of 50–60% and median survival of 18–24 months, though surgical morbidity may delay systemic therapy. Brentuximab vedotin (BV), an anti-CD30 antibody–drug conjugate, combined with CHP (cyclophosphamide, doxorubicin, prednisone), demonstrates superior efficacy in CD30 + lymphomas, with a 68% CR rate and estimated median survival exceeding 24 months in the ECHELON-2 trial, while avoiding vincristine-related neurotoxicity [15–17]. For younger, fit patients, consolidative autologous stem cell transplantation (ASCT) after induction chemotherapy improves 5-year progression-free survival to 61% versus 23% with chemotherapy alone. Therefore, treatment selection must balance disease extent, patient fitness, and CD30 expression, with BV-based regimens offering promise for broader applicability and improved tolerability [18, 19].

In the index case, the patient underwent surgical resection (jejunal resection with J-J anastomosis) due to pneumoperitoneum. It is pertinent to note that while our patient required surgery due to acute complications, previous cases, such as those reported by Kim et al., utilized endoscopy for diagnosis in less symptomatic patients, highlighting non-surgical diagnostic options for earlier detection. They concluded that endoscopically, primary gastrointestinal T-cell lymphomas can be categorized into six major types, with the ulceroinfiltrative form being the most prevalent. Lesions located in the small intestine and those exhibiting an infiltrative pattern were more frequently associated with perforation, contributing to poorer clinical outcomes [3]. Following surgery, the patient was started on BV-CHP chemotherapy for six cycles with G-CSF support. The adjuvant chemotherapeutic regimen of BV-CHP was selected over conventional CHOP based on the strong and diffuse CD30 expression in the neoplastic cells, the patient's young age and good performance status, and emerging evidence from the ECHELON-2 trial demonstrating superior progression-free survival with this regimen in CD30-positive peripheral T-cell lymphomas. As mentioned earlier, elimination of vincristine from the regimen also avoided potential overlapping neurotoxicity with Brentuximab vedotin, particularly important given the aggressive treatment approach required for this high-risk disease [2]. Additionally, G-CSF was administered prophylactically to prevent chemotherapy-induced neutropenia. It has been six months since the patient was initially diagnosed. Over this period, the patient has completed five cycles of systemic chemotherapy. A PET scan conducted after the fourth cycle revealed no evidence of metabolically active disease. Given the excellent treatment response, ASCT is not deemed necessary at this stage.

## Conclusion

Primary ALK-negative ALCL of the small intestine is extremely rare, with fewer than 20 cases reported globally. Our case contributes to this limited literature and highlights the diagnostic and therapeutic challenges in managing this aggressive entity. A high index of suspicion is essential when evaluating T-cell lymphomas of the small intestine, given their aggressive nature. Accurate diagnosis requires a multidisciplinary approach, incorporating radiological imaging, meticulous histopathological examination, and comprehensive immunohistochemical workup. This collaborative strategy is critical for ensuring optimal management and improved outcomes for affected patients.

## Conflict of interests

The authors declare no competing interests.

## Data Availability

Data sharing is not applicable to this article as no datasets were generated or analyzed during the study.
